# Synthesis and Combination Studies of Novel Dipeptide Nitriles with Curcumin for a Potent Synergistic Action Against Rhodesain, Cysteine Protease of *Trypanosoma brucei rhodesiense*

**DOI:** 10.3390/ph18060847

**Published:** 2025-06-05

**Authors:** Carla Di Chio, Josè Starvaggi, Santo Previti, Fabiola De Luca, Benito Natale, Sandro Cosconati, Tanja Schirmeister, Maria Zappalà, Roberta Ettari

**Affiliations:** 1 Department of Chemical, Biological, Pharmaceutical and Environmental Sciences, University of Messina, Viale Ferdinando Stagno d’Alcontres 31, 98166 Messina, Italy; cdichio@unime.it (C.D.C.); jose.starvaggi@studenti.unime.it (J.S.); spreviti@unime.it (S.P.); fabiola.deluca@studenti.unime.it (F.D.L.); mzappala@unime.it (M.Z.); 2 Department of Environmental, Biological and Pharmaceutical Sciences and Technologies, University of Campania Luigi Vanvitelli, Via A. Vivaldi 43, 81100 Caserta, Italy; benito.natale@unicampania.it (B.N.); sandro.cosconati@unicampania.it (S.C.); 3 Institute of Pharmacy and Biochemistry, University of Mainz, Staudingerweg 5, DE 55128 Mainz, Germany; schirmei@uni-mainz.de

**Keywords:** rhodesain, drug combination, nitrile peptides, curcumin, synergistic effect

## Abstract

**Background/Objectives:** Rhodesain is a cysteine protease crucial for the life cycle of *Trypanosoma brucei rhodesiense*, a parasite that causes the lethal form of human African trypanosomiasis. For these reasons, rhodesain is considered an important target for the drug discovery process of novel antitrypanosomal agents. **Methods:** In the present work, we carried out a combination study of two novel synthetic nitriles, **Nitrile 1** and **Nitrile 2**, with curcumin, the golden multitarget nutraceutical obtained from *Curcuma longa* L., which we demonstrated to inhibit rhodesain in a non-competitive manner. We calculated the combination index (CI) in both the combination studies by using the Chou and Talalay method. **Results:** Comparing the CI values of the combinations **Nitrile 1** + curcumin and **Nitrile 2** + curcumin, we assessed that the inhibitory effect of the combination **Nitrile 2** + curcumin against rhodesain was much more potent than that of the other combination. At the IC_50_ value, in the case of the combination **Nitrile 1** + curcumin an additive effect occurred, while in the case of **Nitrile 2 +** curcumin, we observed a moderate synergism: at 99% of the effect, the synergism induced by the combination **Nitrile 2 +** curcumin was much stronger than the synergism promoted by the combination **Nitrile 1** + curcumin (CI = 0.3843 *vs* 0.6622, respectively). **Conclusions:** The co-administration of dipeptide nitriles with curcumin enhances rhodesain inhibition through synergistic effects. Notably, **Nitrile 2** + curcumin exhibits a stronger synergy at higher inhibition levels, indicating a greater therapeutic potential.

## 1. Introduction

Neglected tropical diseases (NTDs) include 20 diseases caused by different pathogens, such as viruses, bacteria, and protozoa, which are particularly prevalent in tropical zones. The World Health Organization (WHO) notes these diseases are prevalent in areas characterized by poor conditions. One of the goals of the WHO is to eradicate and control 20 diseases by 2030 [1]. Human African trypanosomiasis (HAT), also known as sleeping sickness, belongs to the NTDs group and is widespread in Sub-Saharan Africa. It is caused by a parasite of the *Trypanosoma* genus. In particular, there are two subspecies of parasite: *Trypanosoma brucei gambiense*, which is diffused in western and central Africa and is responsible for the chronic form of the disease; and *Trypanosoma brucei rhodesiense*, which in eastern and southern Africa is responsible for the acute form of HAT with a rapid progression [2]. Human transmission is mediated by the bite of blood-feeding tsetse flies belonging to the *Glossina* genus. Sleeping sickness is typically described in two different stages: the hemolymphatic stage, characterized by non-specific symptoms such as fever, weakness, and muscle pain. If the parasite is able to cross the blood–brain barrier (BBB), it can induce the neurological stage which, if not treated in time, can lead to coma and death of the patient [3,4,5].

Only five drugs are available for the treatment of HAT: pentamidine and suramine for the first stage, and melarsoprol, eflornithine, and nifurtimox for the second one. In particular, the nifurtimox-eflornithine combination therapy (NECT) is used for the treatment of the second stage of gambiense HAT [6]. In 2019, fexinidazole, a 5-nitroimidazole, was approved by the EMA for the treatment of HAT in endemic countries. It is the first orally administered drug and effective against gambiense HAT; however, recently WHO expanded the indications of fexinidazole to rhodesiense HAT even if, given the limited data, all recommendations are based on very low-certainty evidence. Furthermore, pediatric patients who do not meet the established age or weight criteria are excluded from fexinidazole therapy [7,8].

The small number of drugs available for the treatment of trypanosomiasis, which present several problems related to toxicity, the mainly parenteral administration, and the limited spectrum of action, encouraged the research towards the identification of new targets for the development of new antitrypanosomal agents. In this context, rhodesain, a cysteine protease of *T. brucei rhodesiense*, represents a promising molecular target for the development of novel therapeutic strategies for HAT treatment [9,10]. Due to its several functions, rhodesain is involved in the disease progression by allowing the penetration of the host blood–brain barrier [11,12]; it also contributes to the survival of the parasite by promoting the evasion of host immune responses by participating in the turnover of the variant surface glycoprotein of the *Trypanosoma* coat [13,14]; finally, it shows relevant proteolytic activity in lysosomes, in the proteolytic degradation of both host proteins intracellularly transported and parasite proteins [15]. Considering all these functions, rhodesain has been identified as a key target for the development of new broad spectrum antitrypanosomal agents.

**Figure 1:**
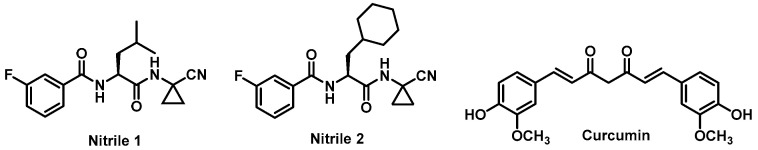
Structure of **Nitrile 1**, **Nitrile 2**, and curcumin.

In recent years, our research group has been focused on the design, synthesis, and biological evaluation of novel rhodesain inhibitors [16]. Recently, new dipeptide nitriles have been developed, endowed with a *K*_i_ value in the nanomolar range towards rhodesain, and they were considered a new starting point for the development of new de-rivatives [17]. In particular, the new compounds bear a cyclopropylcarbonitrile at the P1 site, and at the P2 site a leucine (i.e., **Nitrile 1**) or a cyclohexylalanine (i.e., **Nitrile 2**) residue, which are both preferential for the inhibition of rhodesain (Figure 1).

**Figure 2:**
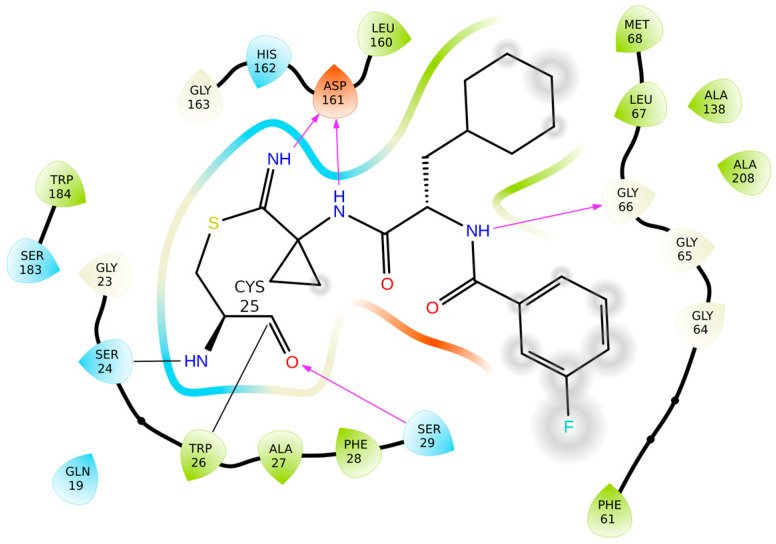
Two-dimensional representation of the predicted binding mode of **Nitrile 2** in complex with rhodesain (PDB ID: 2P86). Only residues within 4 Å of the ligand are shown. H-bonds are depicted as magenta arrows. Docking was attained following the methodology reported elsewhere [17].

The nitrile warhead reversibly reacts with the catalytic cysteine, forming a thioimidate adduct. Moreover, preliminary docking experiments allow us to infer that these nitriles can establish several hydrogen bonds and hydrophobic interactions, with the residues lining the binding site of the enzyme (Figure 2).

The amino group of the P2 residue has been functionalized in both compounds, with a 3-fluorobenzoyl group spanning into the S3 region of the enzyme.

Combination therapy of two synthetic drugs or a synthetic drug combined with a nutraceutical is at present a promising approach for the treatment of various diseases such as cancer, heart, and infectious diseases [18,19,20,21,22]. In particular, there is an increase in the use of nutraceuticals in therapy, which can lead to many advantages; as a matter of fact, combining a nutraceutical with a synthetic inhibitor could improve the treatment efficacy, reduce its toxicity by using a lower dosage of each drug, and decrease the occurrence of resistance. In this study, we selected curcumin as nutraceutical based on its well-documented multitarget pharmacological profile, including anti-inflammatory, antioxidant, and antiparasitic activities, which are particularly relevant in the context of NTDs such as HAT. Our research group demonstrated the ability of curcumin to inhibit rhodesain alone or in combination [23], supporting its potential utility as a complementary agent in rhodesain-targeted therapy. Given its non-toxic nature and its ability to modulate multiple biological pathways, curcumin represents a compelling candidate for combination approaches designed to enhance therapeutic efficacy while potentially reducing drug dosage and associated toxicity.

In addition, recent advances in formulation technologies, including nanoparticle-based delivery systems, liposomal encapsulation, and co-administration with bioenhancers like piperine, have significantly improved curcumin’s pharmacokinetic properties and systemic exposure [24,25]. These developments increase the translational potential of curcumin-based combinations and justify their continued investigation in preclinical models.

In this paper, we carried out two combination studies comparing **Nitrile 1** and **Nitrile 2** with curcumin, using the Chou and Talalay method [26], to evaluate whether the combination of two inhibitors showed a synergistic or additive effect on rhodesain inhibition.

## 2. Results and Discussion

### 2.1. Chemistry

**Scheme 1:**
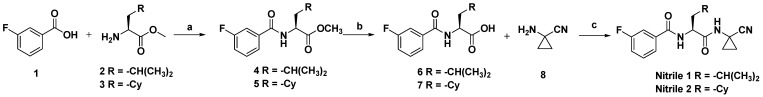
Reagents and conditions: (a) DCM, EDCI, HOBt, 0 °C, then 10 min **2**–**3** DIPEA, rt on; (b) LiOH, MeOH/H_2_O/dioxane (1:1:1), 0 °C, 10 min, then rt, 12h; (c) DCM, EDCI, HOBt, 0 °C, then 10 min **8** DIPEA, rt, 12 h.

For the synthesis of the new dipeptide nitriles, we started from the P3-P2 portion (Scheme 1), the coupling reaction between the 3-fluorobenzoic acid **1** and leucine or cyclo-hexyl alanine methyl ester **2**–**3**, followed by basic hydrolysis resulting in the corresponding carboxylic acids **6**–**7**. The final compounds, **Nitrile 1** and **Nitrile 2**, were then obtained with a coupling reaction between the intermediates **6**–**7** and the cyclopropylcarbonitrile **8**, in the presence of coupling reagents 1-(3-dimethylaminopropyl)-3-ethylcarbodiimide hydrochloride (EDCI) and 1-hydroxybenzotriazole (HOBt). The NMR characterization and signal assignment is reported in the Supplementary Materials.

### 2.2. Biological Evaluation

**Figure 3:**
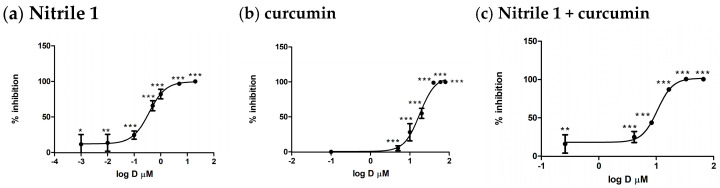
Dose–response curves showing response against rhodesain of **Nitrile 1** (**a**), curcumin (**b**), and **Nitrile 1** + curcumin (**c**). Each experiment was performed in duplicate with * *p* < 0.05, ** *p* < 0.01, *** *p* < 0.001 *vs* no inhibitor.

Now, we describe the combination study comparing **Nitrile 1** and **Nitrile 2** with curcumin separately, to investigate which combination was the most effective for rhodesain inhibition. We tested **Nitrile 1** and curcumin against recombinant rhodesain by using carbobenzyloxy-Phe-Arg 7-amido-4-methylcoumarin hydrochloride (Cbz-Phe-Arg-AMC) as a fluorogenic substrate. Preliminary evaluation at constant inhibitor doses (100 μM, 1 μM, and 0.1 μM) was conducted to determine the range of activity of both **Nitrile 1** and curcumin. Then, Tian continuous assays of **Nitrile 1** and curcumin were performed, each in duplicate. Seven different concentrations were selected, beginning with the lowest dose able to inhibit the enzyme to the highest dose able to fully suppress rhodesain activity. In more detail, we used the range 20 µM − 0.001 for **Nitrile 1**, while 100 µM–1 µM was used for curcumin. The IC_50_ values of both inhibitors were obtained from the analysis of the dose–response curves (Figure 3): 0.26 ± 0.005 µM for **Nitrile 1** and 16.35 ± 0.47 µM for curcumin.

In the subsequent experiment, to determine and evaluate a possible synergistic, additive or antagonistic effect of the combination of the two inhibitors, six concentrations (1/64 × IC_50_, 1/4 × IC_50_, 1/2 × IC_50_, IC_50_, 2 × IC_50_, and 4 × IC_50_, Table 1) were selected to perform the combination assay. Also in this case, the IC_50_ value of the combination was calculated from the dose–response curve, and it was confirmed to be 8.10 ± 0.18 µM (Figure 3c).

**Figure 4:**
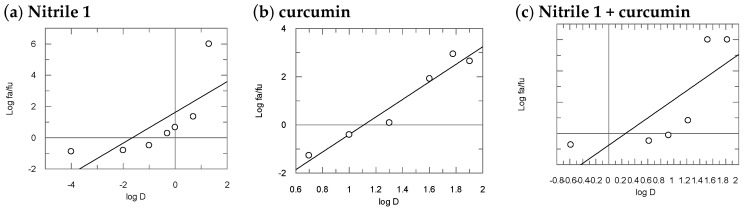
Median effect plot for **Nitrile 1** (**a**), curcumin (**b**), **Nitrile 1** + curcumin (**c**). D is the dose, f_a_ the affected fraction, f_u_ the fraction unaffected by rhodesain.

Subsequently, each dose–response curve was converted into the median effect plot, obtained by plotting the log (f_a_/f_u_) on the y-axis *versus* the log (D) on the x-axis (Figure 4). In this diagram, the maximum point is 1, and not 100, in the dose–response curve; in fact, f_a_ + f_u_ = 1, where f_a_ represents the affected (f_a_), while f_u_ is the unaffected fraction of the enzyme at each dose (D).

The slope of the straight line of each median effect plot is the “m” value. In particular, for **Nitrile 1,** we obtained an m_1_ value of 0.9834, for curcumin a m_2_ value of 3.6394, and for the combination **Nitrile 1** + curcumin an m_1,2_ value of 2.9020, with a **Nitrile 1**/curcumin molar ratio of 1:63. The three m values were calculated using Grafit software (Version 5.0.1.3; Erithacus Software Limited, East Grin-stead, West Sussex, UK), then the doses required to achieve specific percentages of rhodesain inhibition were calculated using the median effect equation [26,27] (Equation (1), see Section 3).

**Figure 5:**
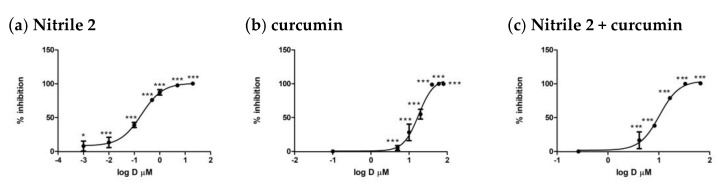
Dose–response curves showing effects against rhodesain of **Nitrile 2** (**a**), curcumin (**b**), **Nitrile 2** + curcumin (**c**). Each experiment was performed in duplicate with * *p* < 0.05, *** *p* < 0.001 vs. no inhibitor.

Then, we followed the same procedure for the combination study of the two inhibitors, **Nitrile 2** and curcumin. Also in this case, a preliminary evaluation at constant inhibitor doses (100 μM, 1 μM, and 0.1 μM) was conducted to determine the range of activity of both **Nitrile 2** and curcumin. Then, we tested **Nitrile 2** and curcumin at seven different concentrations, from 20 to 0.001 µM for **Nitrile 2**, and from 100 to 1 µM for curcumin. The IC_50_ values were calculated by the analysis of dose–response curves (Figure 5), by obtaining 0.14 ± 0.005 µM for **Nitrile 2**, 16.35 ± 0.47 µM for curcumin.

Next, we selected six doses (1/64 × IC_50_, 1/4 × IC_50_, 1/2 × IC_50_, IC_50_, 2 × IC_50_, and 4 × IC_50_, Table 2) to perform the combination assay with **Nitrile 2** + curcumin. The IC_50_ value for the combination was calculated from the dose–response curve (Figure 5c), resulting in 9.42 ± 0.63 µM.

**Figure 6:**
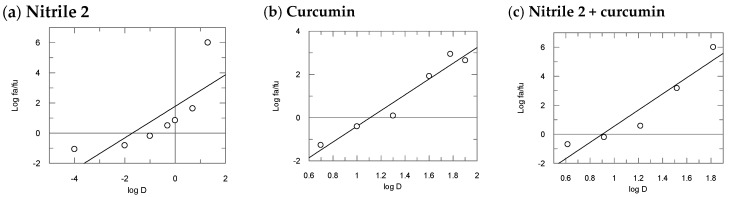
Median Effect Plot for **Nitrile 2** (**a**), curcumin (**b**), **Nitrile 2** + curcumin (**c**). D is the dose, f_a_ the affected fraction, f_u_ the unaffected fraction by rhodesain.

For the second study, the dose–response curves were also converted into the median effect plot (Figure 6), obtaining for **Nitrile 2** an m_1_ value of 1.0440, for curcumin an m_2_ value of 3.6394, and for the combination **Nitrile 2** + curcumin an m_1,2_ value of 5.5770, with a **Nitrile 2**/curcumin molar ratio of 1:117.

The analysis of the m-values and the affected fraction (f_a_) gives useful information about the efficacy and type of interaction between the tested compounds (**Nitrile 1**, **Nitrile 2**, curcumin) and their combinations in relation to rhodesain inhibition. In more detail, **Nitrile 1** (m_1_ = 0.9834) and **Nitrile 2** (m_1_ = 1.0440) showed an m_1_ value close to 1, indicating a sigmoid dose–response curve typical of a reversible inhibition. However, **Nitrile 2** had a slightly higher m_1_, suggesting a marked dose–response curve slope with respect to **Nitrile 1**. Curcumin showed a very high m_2_ (m_2_ = 3.6394), indicating an extremely steep dose–response curve. This means that rhodesain inhibition increased rapidly as the dose changed, making curcumin a particularly potent inhibitor with a sensitive dose-dependent response. The **Nitrile 1** + curcumin combination (m_1,2_ = 2.9020) reduced the slope of the curve compared to single curcumin (m_2_), suggesting that the addition of **Nitrile 1** moderated the extremely steep curcumin response. In contrast, the **Nitrile 2** + curcumin combination (m_1,2_ = 5.5770) resulted in an increased slope compared to single curcumin, indicating that **Nitrile 2** enhanced the dose–response effect of curcumin. This combination was more dynamic and perhaps more effective in terms of interaction.

Considering that **Nitrile 1** and **Nitrile 2** are two competitive inhibitors while curcumin is a non-competitive inhibitor of rhodesain, Chou and Talalay’s method was used for each combination study to investigate the multiple effects of the two drugs. The combination index (CI) was calculated to evaluate the type of interaction in the combined inhibition of rhodesain. It has been established that a combination index (CI) greater than 1 indicates antagonism, a CI equal to 1 represents an additive interaction, and a CI less than 1 denotes synergism [26,27]. The CI was calculated for mutually nonexclusive, independently acting drugs based on Equation (2) (see Section 3).

**Figure 7:**
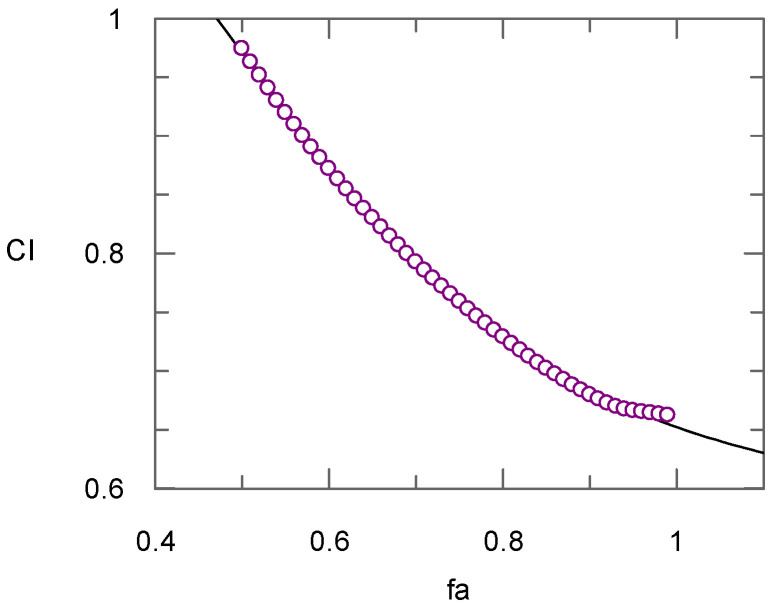
Graphical presentation of the combination index (CI) versus affected fraction (f_a_); i.e., the reduction effect of rhodesain activity obtained from a mixture of **Nitrile 1** and curcumin (molar ratio 1:63).

For the first combination study, **Nitrile 1** + curcumin, starting from the IC_50_, an initial effect of slight synergism was observed, and the CI value was found to be less than 1, in agreement with Chou and Talalay’s rules (Figure 7) [26,27]. It is noteworthy that for the most significant f_a_ values, ranging from 0.6 to 1 (i.e., 60% to 100% inhibition of rhodesain), an increasing synergistic effect was found for the combination **Nitrile 1** + curcumin (Table 3).

**Figure 8:**
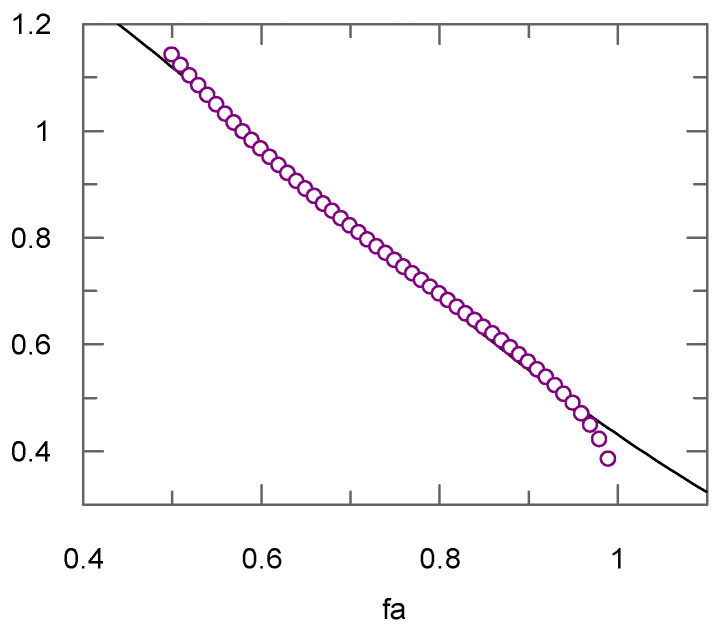
Graphical presentation of the combination index (CI) versus the affected fraction (f_a_); i.e., the reduction effect of rhodesain activity obtained from a mixture of **Nitrile 2** and curcumin (molar ratio 1:117).

The second combination study, involving **Nitrile 2** + curcumin, was calculated as shown above using Grafit software. From the analysis of the obtained results, an initial additive effect was observed; the CI value was found to be equal to 1, in agreement to Chou and Talalay’s rules (Figure 8) [26,27]. Interestingly, for the most significant f_a_ values, ranging from 0.6 to 1 (i.e., 60% to 100% inhibition of rhodesain), an increasing synergistic effect was found for the combination of **Nitrile 2** + curcumin (Table 4).

A comparative evaluation of the **Nitrile 1**–curcumin and **Nitrile 2**–curcumin combinations reveals that both pairs exhibited dose-dependent synergistic effects in rhodesain inhibition, with an increasing synergy observed at higher levels of enzyme inhibition (higher affected fractions). However, a closer inspection of the CI values indicated that the **Nitrile 2**–curcumin combination consistently demonstrated stronger synergy than **Nitrile 1**–curcumin, particularly at higher inhibition levels. At lower affected fractions (fa = 0.5–0.6), the **Nitrile 1** combination showed slight synergism (CI: 0.9744–0.8723), while the **Nitrile 2** combination transitioned from an additive effect (CI: 1.1414 at 50%) to slight synergism (CI: 0.9654 at 60%). As inhibition increases, both combinations showed an improved synergy, but the CI values for **Nitrile 2** were notably lower. At fa = 0.9 and 0.99, **Nitrile 1**–curcumin yielded CI values of 0.6796 and 0.6622, respectively, while **Nitrile 2**–curcumin demonstrated a stronger synergism with CI values of 0.5662 and 0.3843, respectively. These findings suggest that **Nitrile 2**–curcumin is the more synergistic combination, especially at higher inhibition levels, and may offer a greater potential for therapeutic application in rhodesain-targeted treatments. The enhanced synergy observed with **Nitrile 2** may be attributed to little structural differences that promote a more complementary binding or cooperative mechanisms with curcumin at the enzymatic level. Further studies will be necessary to elucidate these molecular interactions and validate their translational potential.

## 3. Materials and Methods

### 3.1. Rhodesain Inhibition Assays

A preliminary screening against rhodesain was carried out using inhibitor concentrations of 100 µM, 1 µM, and 0.1 µM, with an equivalent volume of DMSO as a negative control. The enzyme was recombinantly expressed according to our previously reported protocol [28]. Substrate hydrolysis (Cbz-Phe-Arg-AMC, 10 µM) was continuously monitored for 10 min at room temperature by measuring product release. The assay buffer contained 50 mM sodium acetate, pH = 5.5, 5 mM EDTA, 200 mM NaCl, and 0.005% Brij, a nonionic polyoxyethylene surfactant, to avoid aggregation and wrong positive results. The enzyme buffer contained 5 mM dithiothreitol (DTT) rather than Brij. In the various assays, **Nitrile 1**, **Nitrile 2**, and curcumin were tested separately twice in duplicate in 96-well plates (BRAND^®^, Wertheim, Germany) in a total volume of 200 µL. The following concentrations were used: (i) for **Nitrile 1** and for **Nitrile 2**, 0.001 μM, 0.01 μM, 0.1 μM, 0.5 μM, 1 μM, 5 μM, and 20 μM; and (iii) for curcumin, 0.1 µM, 5 μM, 10 μM, 20 μM, 40 μM, 60 μM, and 80 μM.

Fluorescence of the product AMC of the substrate hydrolyses was measured using an Infinite 200 PRO microplate reader (Tecan, Männedorf, Switzerland) at room temperature with a 380 nm excitation filter and a 460 nm emission filter. Results are expressed as IC_50_ ± SD values and were calculated by fitting progress curves to the four-parameter IC_50_ equation, using the software GraphPad Prism 5.0.3 (GraphPad Software Inc., San Diego, CA, USA).

$$y=\frac{y_{max}-y_{min}}{1+{\left(\frac{\left[I\right]}{{IC}_{50}}\right)}^{S}}+y_{min}$$

where y [∆F/min] represents the substrate hydrolysis rate, y_max_ the maximum value of the dose–response curve measured at inhibitor concentration [I] = 0 µM, y_min_ the minimum value obtained at the highest inhibitor concentration, and s the Hill coefficient.

### 3.2. Calculation of the Combination Index

For the calculation of the combination index of the combination **Nitrile 1** or **Nitrile 2** + curcumin, six data points were used: 1/64 × IC_50 F1+F2_, 1/4 × IC_50 F1+F2_, 1/2 × IC_50 F1+F2_, IC_50 F1+F2_, 2 × IC_50 F1+F2_, and 4 × IC_50 F1+F2_, where F_1_ = **Nitrile 1** or **Nitrile 2** while F_2_ = curcumin (Table 1 and Table 2). After the determination of IC_50_ values ± SD for the drug combination, dose–response curves were converted into median effect plots, normalizing the maximum response to 1 rather than 100. The inhibited fraction of the enzyme was designated as the “affected fraction” (f_a_), while the uninhibited fraction was the “unaffected fraction” (f_u_), with the relationship f_a_ + f_u_ = 1. The median effect plot was generated by plotting log(f_a_/f_u_) against log(D) on the x-axis, allowing the determination of the m value, which corresponded to the Hill coefficient and reflected the sigmoidal nature (S-shaped curve) of the dose–response relationship.

Once the three different m values were calculated by Grafit software (Version 5.0; Erithacus Software Limited, East Grinstead, West Sussex, UK), we established the single doses able to inhibit the enzyme for a specific percentage of inhibition by means of the median effect equation [26,27]:

D = IC_50_ [f_a_/f_u_]^1/m^ (1)

The Chou–Talalay method was then applied to evaluate multiple drug effects [26,27]. The CI for mutually exclusive drugs, which act independently, was calculated as follows:

CI = [(D)_1_/(IC_50_)_1_]+[(D)_2_/(IC_50_)_2_] + [(D)_1_(D)_2_]/[(IC_50_)_1_(IC_50_)_2_] (2)

where (IC_50_)_1_ and (IC_50_)_2_ correspond to the doses required to induce 50% inhibition of the enzyme by **Nitrile 1** or **Nitrile 2** and curcumin, respectively, while D_1_ and D_2_ represent the doses of **Nitrile 1** or **Nitrile 2** and curcumin capable of inducing a specific percentage inhibition of rhodesain, obtained using the median effect equation, as previously described. The (IC_50_)_1_ and (IC_50_)_2_ were obtained by dose–response curves, and D_1_ and D_2_ by the median effect equation. The Grafit software was used to calculate the CI for rhodesain inhibition percentages from 50% to 100%.

### 3.3. Statistical Analyses

The statistical analysis of the data was performed using the one-way test (ANOVA) with Dunnett’s multiple comparison test, considering significant differences of *p* < 0.05 with respect to the percentage of rhodesain inhibition of curcumin, Nitrile 1, Nitrile 2, curcumin + Nitrile 1, and curcumin + Nitrile 2. The analyses were performed with GraphPad Prism 5.0.3. Results are expressed as the arithmetic mean standard deviation (SD).

## 4. Conclusions

In summary, the results from both combination studies demonstrated that co-administration of dipeptide nitriles with curcumin enhanced rhodesain inhibition, supporting the potential utility of these combinations in antitrypanosomal therapy. Notably, the **Nitrile 2** + curcumin combination exhibited a stronger synergism, particularly at higher affected fractions (fa ≥ 0.80), as evidenced by significantly lower combination index (CI) values compared to the **Nitrile 1** + curcumin combination. These findings suggest that **Nitrile 2** may act in greater concert with curcumin, offering a more potent inhibitory effect at elevated levels of enzymatic activity suppression. Overall, the choice between these combinations may be guided by therapeutic objectives: **Nitrile 1** + curcumin may be preferred when a more moderate and progressive inhibition is desirable, while **Nitrile 2** + curcumin could be advantageous in scenarios requiring maximum efficacy and stronger synergistic effects. These data provide a promising base for further preclinical evaluation and optimization of combination regimens targeting rhodesain in HAT treatment.

## Figures and Tables

**Table 1:** Six selected doses for the combination assay of **Nitrile 1** and curcumin.

	1/64 × IC_50_	1/4 × IC_50_	1/2 × IC_50_	IC_50_	2 × IC_50_	4 × IC_50_
**Nitrile 1**	0.004 μM	0.066 μM	0.13 μM	0.26 μM	0.53 μM	1.06 μM
Curcumin	0.26 μM	4.09 μM	8.18 μM	16.36 μM	32.71 μM	65.42 μM
**Nitrile 1 +** curcumin	0.004 + 0.26 μM	0.066 + 4.09 μM	0.13 + 8.18 μM	0.26 +16.36 μM	0.53 + 32.71 μM	1.06 +65.42 μM

**Table 2:** Six selected doses for the combination assay of **Nitrile 2** and curcumin.

	1/64 × IC_50_	1/4 × IC_50_	1/2 × IC_50_	IC_50_	2 × IC_50_	4 × IC_50_
**Nitrile 2**	0.0022 μM	0.035 μM	0.07 μM	0.14 μM	0.28 μM	0.56 μM
Curcumin	0.26 μM	4.09 μM	8.18 μM	16.36 μM	32.71 μM	65.42 μM
**Nitrile 2 +** curcumin	0.0022+ 0.26 μM	0.035+ 4.09 μM	0.07+ 8.18 μM	0.14+ 16.36 μM	0.28+ 32.71 μM	0.56+ 5.42 μM

**Table 3:** Combination index (CI) values of **Nitrile 1** and curcumin used in combination for the reduction of rhodesain activity.

Affected Fraction (f_a_)	% Rhodesain Inhibition	CI	Diagnosis CI
0.5	50%	0.9744	Slight synergism
0.6	60%	0.8723	Slight synergism
0.7	70%	0.7927	Moderate synergism
0.8	80%	0.7289	Moderate synergism
0.9	90%	0.6796	Synergism
0.99	99%	0.6622	Synergism

**Table 4:** Combination index values of **Nitrile 2**-curcumin used in combination for the reduction of rhodesain activity.

Affected Fraction (f_a_)	% Rhodesain Inhibition	CI	Diagnosis CI
0.5	50%	1.1414	Additive
0.6	60%	0.9654	Slight synergism
0.7	70%	0.8216	Moderate synergism
0.8	80%	0.6942	Synergism
0.9	90%	0.5662	Synergism
0.99	99%	0.3843	Synergism

## Data Availability

The original contributions presented in the study are included in the article, further inquiries can be directed to the corresponding author.

## Supplementary Materials

The following supporting information can be downloaded at https://www.mdpi.com/article/10.3390/ph18060847/s1, Figure S1: ^1^H NMR spectrum of compound **5**. Figure S2: ^13^C NMR spectrum of compound **5**. Figure S3: ^1^H NMR spectrum of compound **7**. Figure S4: ^1^H NMR spectrum of compound **Nitrile 1**. Figure S5: ^13^C NMR spectrum of compound **Nitrile 1**. Figure S6: ^1^H NMR spectrum of compound **Nitrile 2**. Figure S7: ^13^C NMR spectrum of compound **Nitrile 2**.

## Abbreviations

The following abbreviations are used in this manuscript:

NTDs	Neglected tropical diseases
WHO	World Health Organization
HAT	Human African trypanosomiasis
BBB	Blood–brain barrier
NECT	Nifurtimox-eflornithine combination
EMA	European Medicines Agency
Cbz	Benzyloxycarbonyl
AMC	Aminomethylcumarin
