# Supplementary material for: Synthesis and Combination Studies of Novel Dipeptide Nitriles with Curcumin for a Potent Synergistic Action Against Rhodesain, Cysteine Protease of Trypanosoma brucei rhodesiense
Source: Pharmaceuticals (Basel). 2025 Jun 5;18(6):847. doi: 10.3390/ph18060847 (PMC12195826; doi:10.3390/ph18060847)
Supplement: Supplementary file 1 [file pharmaceuticals-18-00847-s001.zip › pharmaceuticals-3653940-supplementary.pdf]

Supporting information

Synthesis and combination studies of novel dipeptide nitriles with curcumin for a potent synergistic action against rhodesain, cysteine protease of *Trypanosoma brucei rhodesiense*

Carla Di Chio[1†], Josè Starvaggi[1†], Santo Previti[1], Fabiola De Luca[1], Benito Natale[2], Sandro Cosconati[2], Tanja Schirmeister[3], Maria Zappalà[1] and Roberta Ettari[1*]

1 Department of Chemical, Biological, Pharmaceutical and Environmental Sciences, University of Messina, Viale Ferdinando Stagno d'Alcontres 31, 98166 Messina, Italy; cdichio@unime.it (C.D.C.); jose.starvaggi@studenti.unime.it (J.S.); spreviti@unime.it (S.P.); fabiola.deluca@studenti.unime.it (F.D.L.); mzappala@unime.it (M.Z.)

2 Department of Environmental, Biological and Pharmaceutical Sciences and Technologies, University of Campania Luigi Vanvitelli, Via A. Vivaldi 43, 81100 Caserta, Italy; benito.natale@unicampania.it (B.N.); sandro.cosconati@unicampania.it (S.C.)

3 Institute of Pharmacy and Biochemistry, University of Mainz, Staudingerweg 5, DE 55128 Mainz, Germany; schirmei@uni-mainz.de

* Correspondence: rettari@unime.it; Tel.: +39-090-676-6554

† These authors equally contributed to this work.

Experimental section

Chemistry

All reagents and solvents were obtained from commercial suppliers and were used without further purification. Elemental analyses were carried out on a C. Erba Model 1106 (Elemental Analyser for C, H, and N) instrument, and the obtained results are within ±0.4% of the theoretical values. Merck silica gel 60 F254 plates were used for analytical TLC; flash column chromatography was performed on Merck silica gel (200–400 mesh). ^{1}H and ^{13}C and NMR spectra were recorded on a Varian 500 MHz spectrometer equipped with a ONE_NMR probe and operating at 499.74 and 125.73 MHz for ^{1}H and ^{13}C, respectively. We used the residual signal of the deuterated solvent as an internal standard. Splitting patterns are described as singlet (s), doublet (d), doublet of doublet (dd), triplet (t), quartet (q), multiplet (m), or broad singlet (bs). ^{1}H and ^{13}C NMR chemical shifts (δ) are expressed in ppm and coupling constants (*J*) are given in Hz.

Synthesis of dipeptide nitriles 1-2

Methyl 2-(3-fluorobenzamido)-4-methylpentanoate (4)

To a solution of 3-fluorobenzoic acid **1** (92.55 mg, 0.66 mmol) in dry DCM, HOBt (111.55 mg, 0.82 mmol) and EDCI (158.29 mg, 0.82 mmol) were added at 0° C. After 10 min, the methyl 2-amino-4-methylpentanoate hydrochloride **2** (100 mg, 0.55 mmol) and DIPEA (195.66 µl, 1.10 mmol) were added and the reaction mixture was stirred at room temperature overnight. After this time, the reaction mixture was washed with brine, dried over Na_2SO_4, filtered and concentrated *in vacuo*. The crude residue was purified by column chromatography using light petroleum/ EtOAc (8:2) to obtain coupling product **6** (132.4 mg, 90%). Consistency: white powder; R_f = 0.32 (light petroleum/ EtOAc 8:2). The spectroscopic data are in agreement with those reported in literature.[1]

Methyl 2-(3-fluorobenzamido)-3-cyclohexylpropanoate (5)

To a solution of 3-fluorobenzoic acid **1** (90.8 mg, 0.65 mmol) with the same procedure described for compound **4**, we obtained the crude residue, which was purified by column chromatography using light petroleum/ EtOAc (85:15) to obtain coupling product **5** (96 mg, 58%). Consistency: white oil; R_f = 0.60 (light petroleum/ EtOAc 8:2); ^{1}H NMR (500 MHz, $CDCl_3$) = *δ*: 0.86 – 1.01 (m, 2H), 1.08 – 1.25 (m, 3H), 1.33 – 1.43 (m, 1H), 1.58 – 1.72 (m, 5H), 1.72 – 1.79 (m, 1H), 1.82 (d, *J* = 12.6 Hz, 1H), 3.75 (s, 3H), 4.79 – 4.87 (m, 1H), 6.79 (t, *J* = 13.3 Hz, 1H), 7.13 – 7.19 (m, 1H), 7.31 – 7.40 (m, 1H), 7.48 (d, *J* = 5.7 Hz, 1H), 7.53 (d, *J* = 7.7 Hz, 1H) ppm; ^{13}C NMR (75 MHz, $CDCl_3$) = *δ:* 26.06,

26.22, 26.41, 32.65, 33.59, 34.33, 40.22, 50.76, 52.53, 114.62 (d, J = 23.0 Hz), 118.76 (d, J = 21.4 Hz), 122.62, 130.26, 136.19, 162.78 (d, J = 247.7 Hz), 166.96, 173.91 ppm.

2-(3-fluorobenzamido)-4-methylpentanoic acid (6)

To a solution of the **4** (132.4 mg, 0.50 mmol) in a mixture mixture methanol/water/dioxane (1:1:1), LiOH as powder (34.8 mg, 1.45 mmol) was added at 0° C. The reaction mixture was stirred at room temperature for 12h. After this time, the solvents were evaporated *in vacuo*. The residue was treated with 10% solution of $KHSO_4$ and the organic phase was extracted with EtOAc, dried over Na_2SO_4 and concentrated to obtained the pure carboxylic acid **6** (119 mg, 95%); Consistency: white powder. The spectroscopic data are in agreement with those reported in literature.[1]

2-(3-fluorobenzamido)-3-cyclohexylpropanoic acid (7)

To a solution of the **5** (96 mg, 0.31 mmol) as the same described procedure for compound **6**, we obtained the pure carboxylic acid **7** (79 mg, 87%); Consistency: white powder; ^{1}H NMR (500 MHz, $CDCl_3$) = δ: 0.88 – 1.05 (m, 2H), 1.09 – 1.29 (m, 3H), 1.37 – 1.42 (m, 1H), 1.61 – 1.76 (m, 5H), 1.79 – 1.88 (m, 2H), 4.79 – 4.86 (m, 1H), 6.76 (d, J = 8.0 Hz, 1H), 7.20 (t, J = 8.0 Hz, 1H), 7.39 (dd, J = 13.5 Hz and 7.7 Hz, 1H), 7.50 (d, J = 9.3 Hz, 1H), 7.55 (d, J = 7.7 Hz, 1H) ppm.

***N*-(1-(1-cyanocyclopropylcarbamoyl)-3-methylbutyl)-3-fluorobenzamide (Nitrile 1)**

To a solution of 2-(3-fluorobenzamido)-4-methylpentanoic acid **6** (119 mg, 0.47 mmol) with the same procedure described for compound **4**, we obtained the crude residue, which was purified by column chromatography using light petroleum/ EtOAc (5:5) to obtain coupling product **Nitrile 1** (85 mg, 57%). Consistency: light yellow powder; R_f = 0.46 (light petroleum/ EtOAc 5:5); ^{1}H NMR (500 MHz, CD_3OD) δ:= 1.00 (dd, J = 15.2 Hz and 6.0 Hz, 6H, $\underline{CH_3}$-CH-$\underline{CH_3}$), 1.25 – 1.30 (m, 2H, -$\underline{CH_2}$-iPr), 1.50 – 1.56 (m, 2H, $\underline{CH_2}$ of Cp), 1.61 – 1.69 (m, 1H, CH_3-$\underline{CH}$-CH_3), 1.72 –1.85 (m, 2H, $\underline{CH_2}$ of Cp), 4.53 – 4.60 (m, 1H, NH-$\underline{CH}$-CO), 7.32 (t, J = 12.3 Hz, 1H, $\underline{CH}$Ar), 7.51 (dd, J = 14.2 Hz and 6.5 Hz, 1H, $\underline{CH}$Ar), 7.61 (d, J = 9.7 Hz, 1H, $\underline{CH}$Ar), 7.71 (d, J = 7.8 Hz, 1H, $\underline{CH}$Ar) ppm; ^{13}C NMR (75 MHz, CD_3OD) = δ: 16.70 ($\underline{CH_2}$ of Cp), 17.10 ($\underline{CH_2}$ of Cp), 21.37 ($\underline{CH_3}$-CH), 21.92 ($\underline{CH_3}$-CH), 23.36 (CH_3-$\underline{CH}$-CH_3), 26.11 ($\underline{Cq}$ of Cp), 41.47 (-$\underline{CH_2}$-iPr), 53.61(NH-$\underline{CH}$-CO), 115.49 (d, J = 23.4 Hz, $\underline{CH}$Ar), 119.60 (d, J = 21.5 Hz, $\underline{CH}$Ar), 121.23 (-$\underline{CN}$), 124.42 ($\underline{CH}$Ar), 131.47 (d, J = 7.9 Hz, $\underline{Cq}$Ar), 137.38 (d, J = 7.0 Hz, $\underline{CH}$Ar), 163.99 (d, J = 245.6 Hz, F-$\underline{Cq}$Ar), 168.85 (Ar-$\underline{CO}$-NH), 176.18 (CH-$\underline{CO}$ -NH) ppm. Elemental analysis: calcd for $C_{17}H_{20}FN_3O_2$: C 64.34, H 6.35, N 13.24; found: C 64.22, H 6.06, N 13.46.

_N_-(1-(1-cyanocyclopropylcarbamoyl)-2-cyclohexylethyl)-3-fluorobenzamide (Nitrile 2)

To a solution of 2-(3-fluorobenzamido)-3-cyclohexylpropanoic acid **7** (79 mg, 0.27 mmol) with the same procedure described for compound **4**, we obtained the crude residue, which was purified by column chromatography using light petroleum/ EtOAc (6:4) to obtain coupling product **Nitrile 2** (36 mg, 38%). Consistency: white powder; R_f = 0.44 (light petroleum/ EtOAc 6:4); ^{1}H NMR (500 MHz, CD_3OD) δ:= 0.95 – 1.09 (m, 2H, *CH₂* of Cy), 1.17 – 1.34 (m, 6H, *CH₂* of Cy), 1.35 – 1.48 (m, 1H, *CH* of Cy), 1.49 – 1.55 (m, 2H, *CH₂* of Cy), 1.63 – 1.71 (m, 2H, *CH₂* of Cp), 1.62 – 1.80 (m, 4H, *CH₂* of Cp & *CH₂*-Cy), 4.54 – 4.60 (m, 1H, NH-*CH*-CO), 7.31 (t, *J* = 8.4 Hz, 1H, *CH*Ar), 7.51 (dt, *J* = 13.8 Hz and 3.9 Hz, 1H, *CH*Ar), 7.61 (d, *J* = 9.7 Hz, 1H, *CH*Ar), 7.70 (d, *J* = 7.8 Hz, 1H, *CH*Ar) ppm; ^{13}C NMR (75 MHz, CD_3OD) = δ: 16.97 (CH_2 of Cp), 17.42 (CH_2 of Cp), 21.67 (*Cq* of Cp), 27.52 (CH_2 of Cy), 27.68 (CH_2 of Cy), 27.83 (CH_2 of Cy), 33.73 (CH of Cy), 35.03 (CH_2 of Cy), 35.93 (CH_2 of Cy), 40.39 (-CH_2-Cy), 53.26 (NH-CH-CO), 115.80 (d, *J* = 23.3 Hz, CHAr), 119.91 (d, *J* = 21.5 Hz, CHAr), 121.52 (-CN), 124.75 (CHAr), 131.79 (d, *J* = 7.9 Hz, CHAr), 137.69 (*Cq*Ar), 164.32 (d, *J* = 245.7 Hz, F-*Cq*Ar), 169.13 (Ar-CO-NH), 176.58 (CH- CO -NH) ppm. Elemental analysis: calcd for $C_{20}H_{24}FN_3O_2$: C 67.21, H 6.77, N 11.76; found: C 66.98, H 6.49, N 11.96.

Figure S1: ^{1}H NMR spectrum of compound **5**.

Figure S2: ^{13}C NMR spectrum of compound **5**.

Figure S3: ^{1}H NMR spectrum of compound **7**.

Figure S4: ^{1}H NMR spectrum of compound **Nitrile 1**.

Figure S5: ^{13}C NMR spectrum of compound **Nitrile 1**.

Figure S6: ^{1}H NMR spectrum of compound **Nitrile 2**.

Figure S7: ^{13}C NMR spectrum of compound **Nitrile 2**.

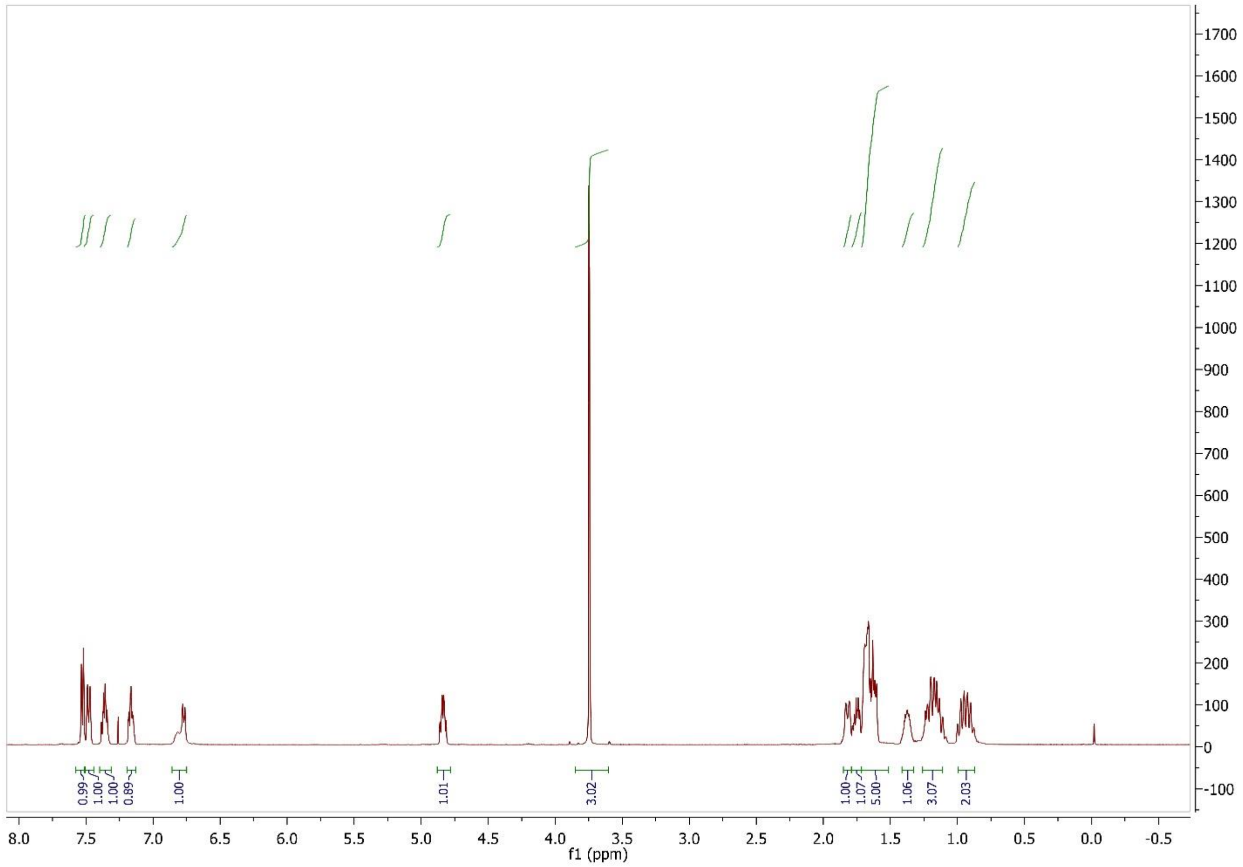

Figure S1: ^{1}H NMR spectrum of compound **5**.

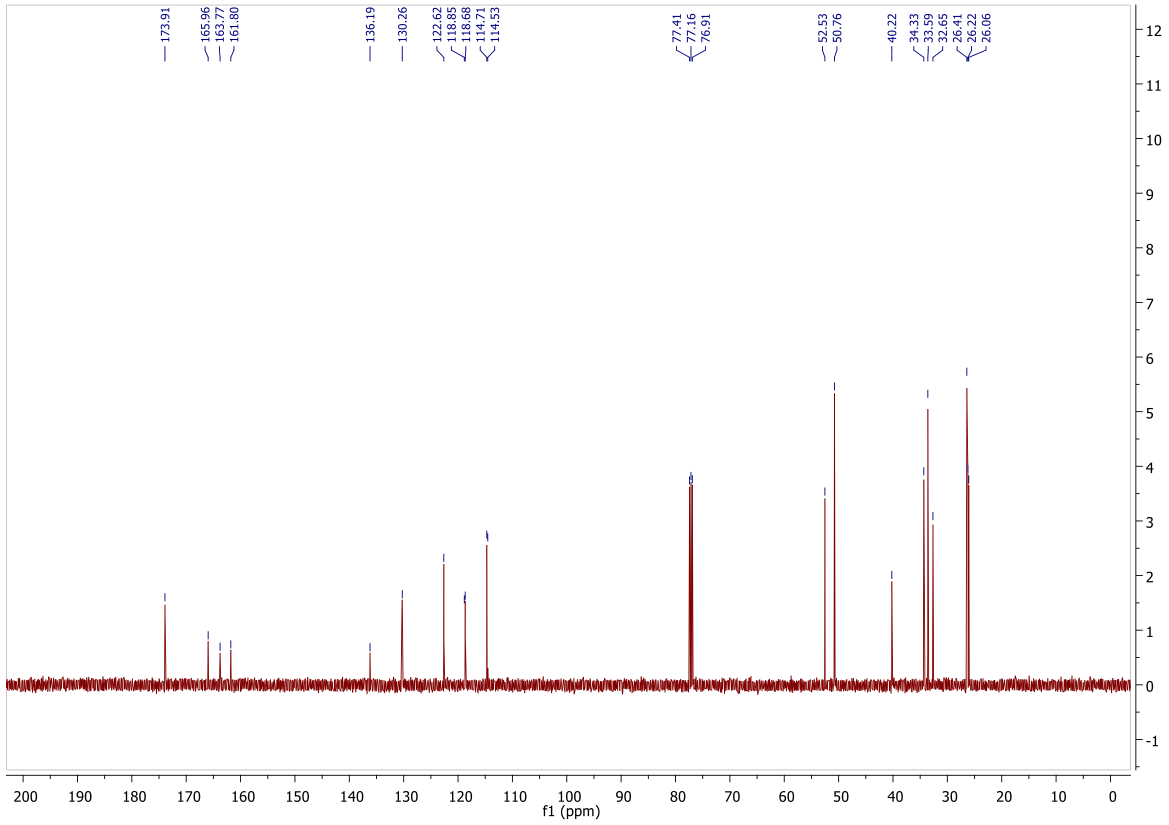

Figure S2: ^{13}C NMR spectrum of compound **5**.

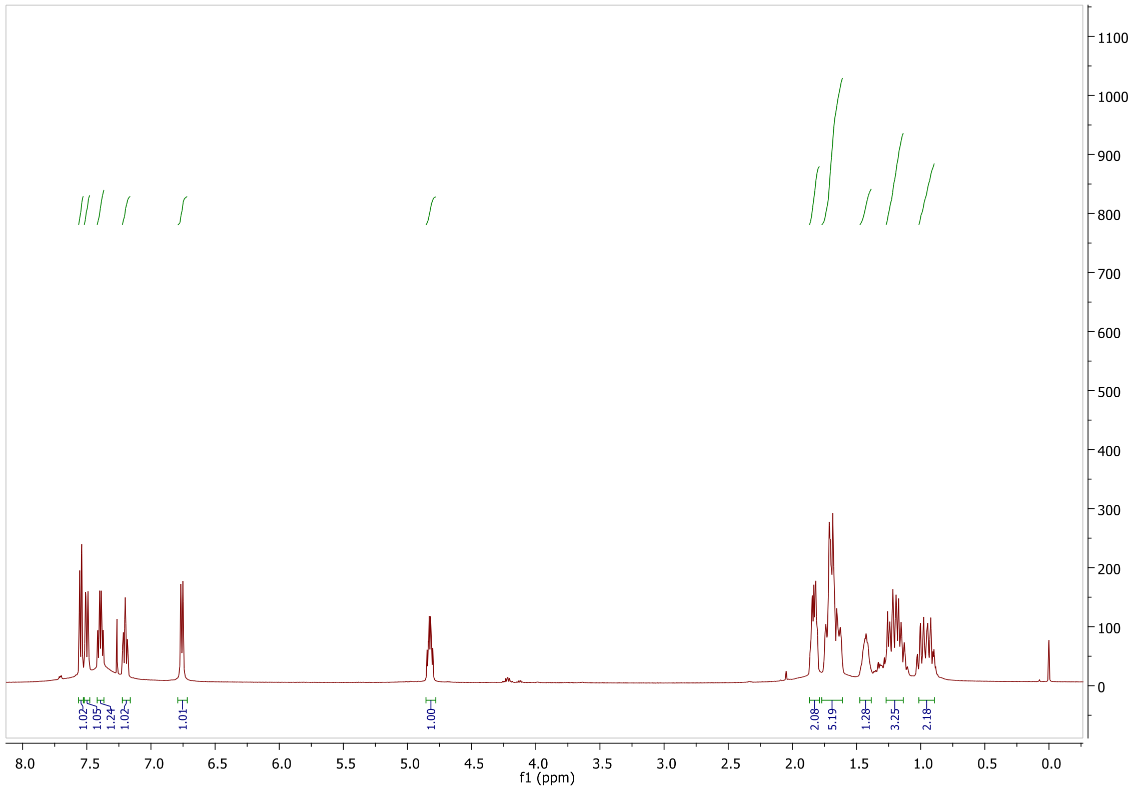

Figure S3: ^{1}H NMR spectrum of compound **7**.

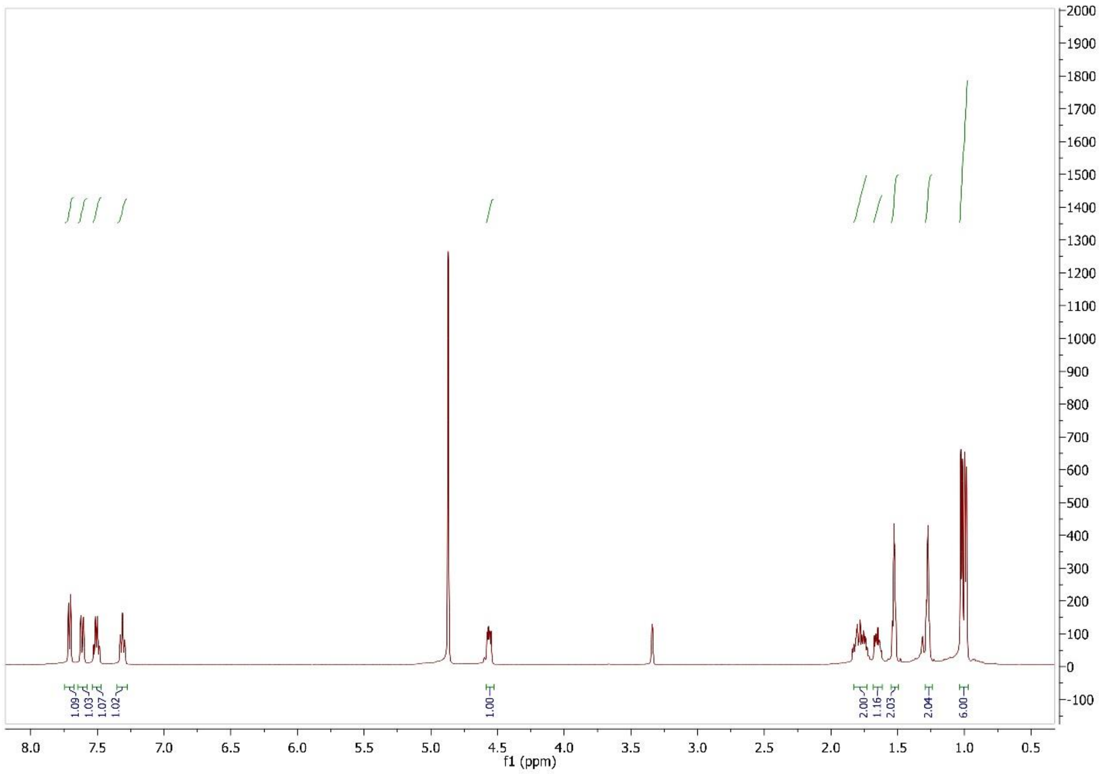

Figure S4: ^{1}H NMR spectrum of compound **Nitrile 1**.

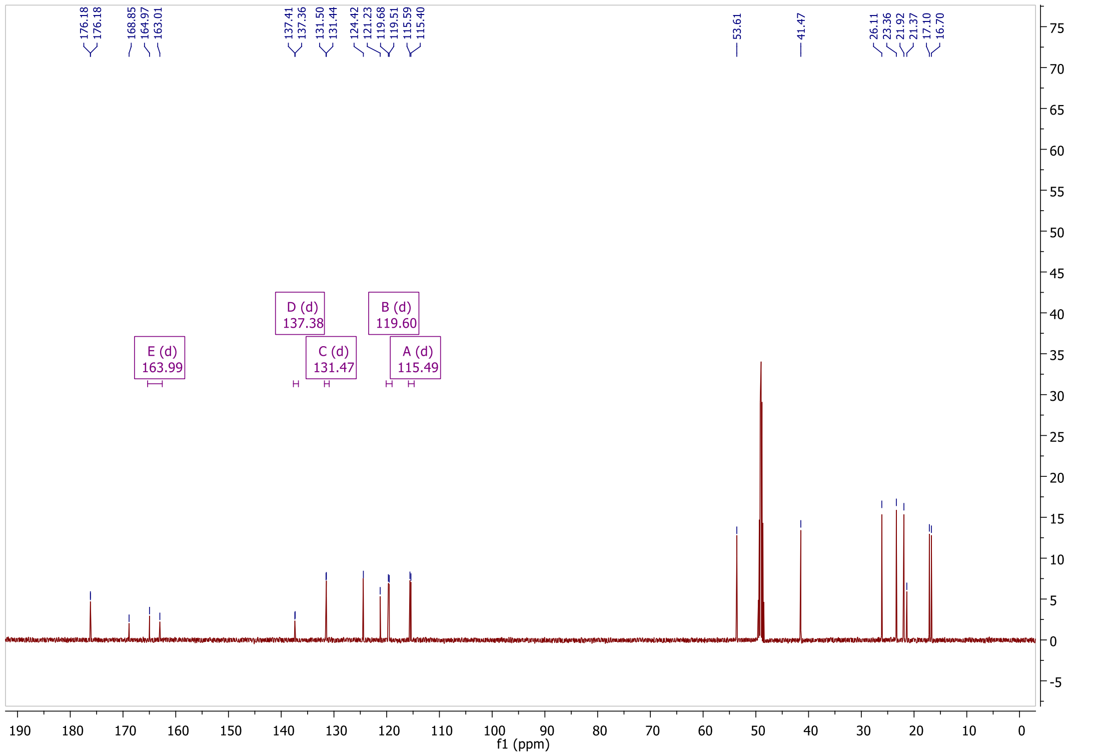

Figure S5: ^{13}C NMR spectrum of compound **Nitrile 1**.

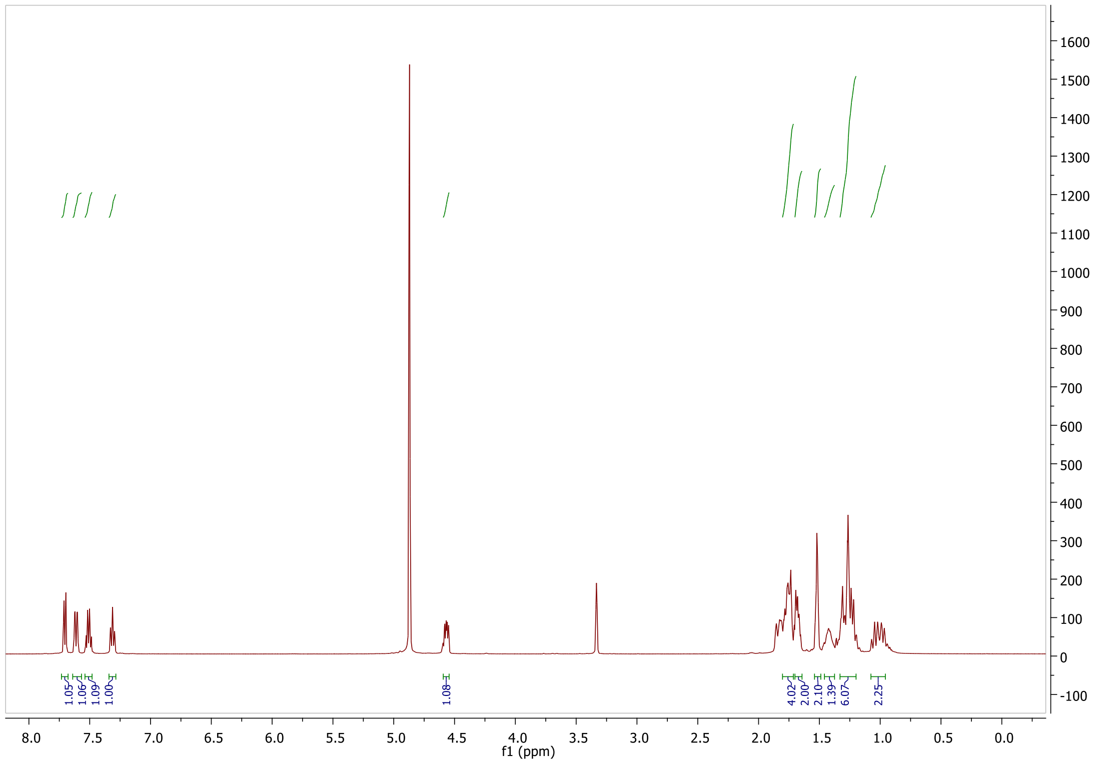

Figure S6: ^{1}H NMR spectrum of compound **Nitrile 2**.

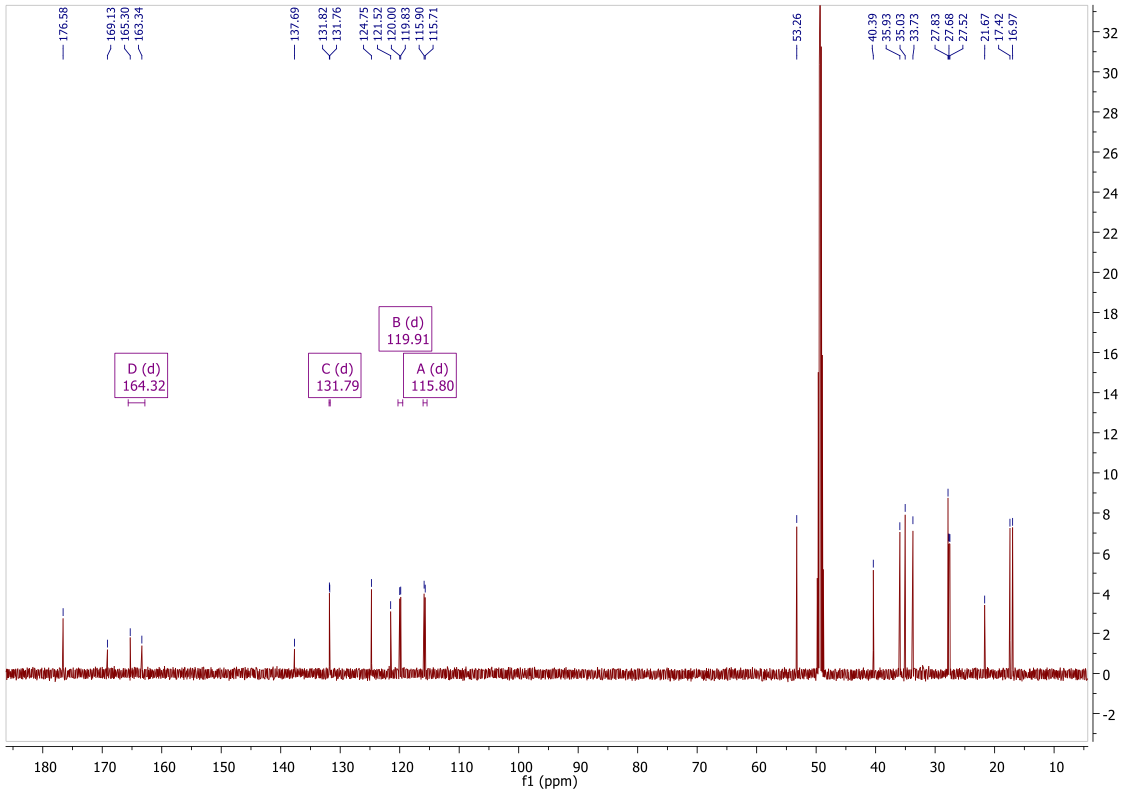

Figure S7: ^{13}C NMR spectrum of compound **Nitrile 2**.

References:

1. Di Chio, C.; Previti, S.; Amendola, G.; Ravichandran, R.; Wagner, A.; Cosconati, S.; Hellmich, U.A.; Schirmeister, T.; Zappalà, M.; Ettari, R. Development of novel dipeptide nitriles as inhibitors of rhodesain of Trypanosoma brucei rhodesiense. *European J Med. Chem.* **2022**, *236*, 114328.
